# Advances in Regenerative Dentistry: A Systematic Review of Harnessing Wnt/β-Catenin in Dentin-Pulp Regeneration

**DOI:** 10.3390/cells13131153

**Published:** 2024-07-06

**Authors:** Mariam Amir, Lakshmi Jeevithan, Maham Barkat, Syeda Habib Fatima, Malalai Khan, Sara Israr, Fatima Naseer, Sarmad Fayyaz, Jeevithan Elango, Wenhui Wu, José Eduardo Maté Sánchez de Val, Saeed Ur Rahman

**Affiliations:** 1 Department of Oral Biology, Institute of Basic Medical Sciences, Khyber Medical University, Peshawar 25000, Pakistan; 2 Department of Marine Biopharmacology, College of Food Science and Technology, Shanghai Ocean University, Shanghai 201306, Chinawhwu@shou.edu.cn (W.W.); 3 Department of Dental Materials Institute of Basic Medical Sciences, Khyber Medical University, Peshawar 25000, Pakistan; 4 Center of Molecular Medicine and Diagnostics (COMManD), Department of Biochemistry, Saveetha Dental College and Hospitals, Saveetha Institute of Medical and Technical Sciences, Saveetha University, Chennai 600077, India; 5 Department of Biomaterials Engineering, Faculty of Health Sciences, UCAM—Universidad Católica San Antonio de Murcia, Guadalupe, 30107 Murcia, Spain; jemate@ucam.edu

**Keywords:** Wnt/β-catenin, signaling pathway, dentin, pulp, regeneration

## Abstract

Dentin pulp has a complex function as a major unit in maintaining the vitality of teeth. In this sense, the Wnt/β-Catenin pathway has a vital part in tooth development, maintenance, repair, and regeneration by controlling physiological activities such as growth, differentiation, and migration. This pathway consists of a network of proteins, such as Wnt signaling molecules, which interact with receptors of targeted cells and play a role in development and adult tissue homeostasis. The Wnt signals are specific spatiotemporally, suggesting its intricate mechanism in development, regulation, repair, and regeneration by the formation of tertiary dentin. This review provides an overview of the recent advances in the Wnt/β-Catenin signaling pathway in dentin and pulp regeneration, how different proteins, molecules, and ligands influence this pathway, either upregulating or silencing it, and how it may be used in the future for clinical dentistry, in vital pulp therapy as an effective treatment for dental caries, as an alternative approach for root canal therapy, and to provide a path for therapeutic and regenerative dentistry.

## 1. Introduction

The tooth is an intricate soft connective tissue, which is surrounded by a combination of hard structures such as enamel, dentin, and cementum. Worldwide, tooth loss and damage are considered to be a global health problem that may be caused by caries, trauma, or iatrogenic factors [1]. The prevalence of traumatic dental injuries in permanent and primary dentition was registered to be 15.2% and 22.7% worldwide, respectively, indicating the high prevalence of dental injury [2]. The dental pulp contributes to the maintenance of tooth function by participating in homeostasis, repair, and regeneration in response to trauma or infection. This process involves the formation of odontoblast-like cells, which deposit a matrix to create reparative dentin [3]. When inflamed, the pulp expresses an intrinsic anti-inflammatory response, with elevated dentin regeneration capacity observed at all ages [4]. Stem cells of pulpal origin are multipotent mesenchymal cells with regenerative abilities [5,6]. They are present in both deciduous and permanent teeth pulp, where they initiate regenerative signals [7,8]. The importance of these cells as regulators of the pulp repair process is perceived by their movement and differentiation at the injury site to form reparative dentin and it can be used in stem cell-based medicinal regeneration and research [9,10].

**Figure 1:**
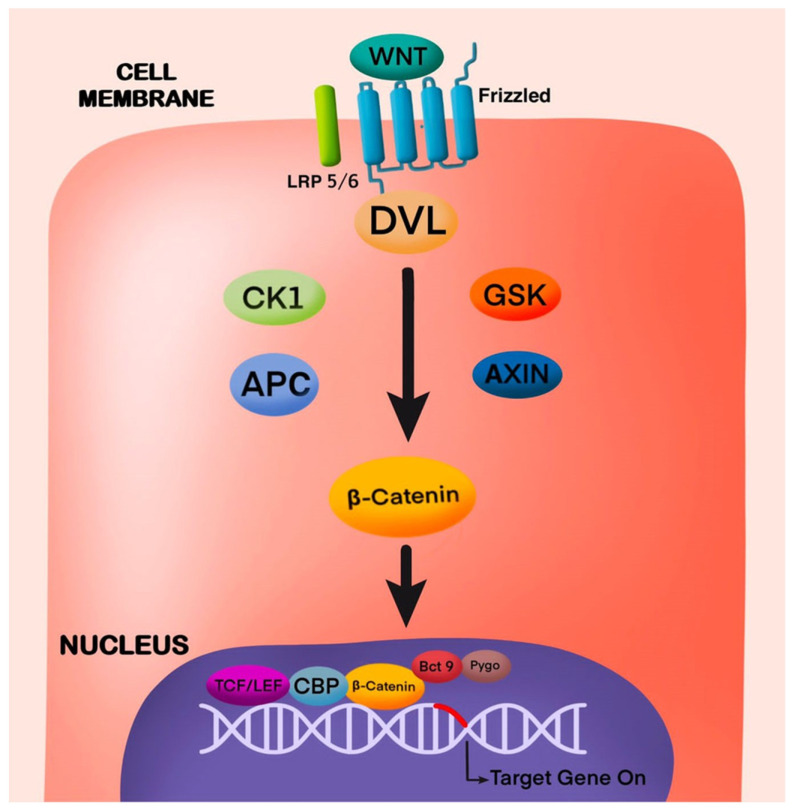
Active Canonical Wnt/β-Catenin Signaling Pathway. Wnt signal triggers Frizzled protein and activates LRP 5/6, Dishevelled (Dvl), Glycogen Synthase Kinase 3β (GSK), Casein kinase 1 (CK1), adenomatous polyposis coli (APC).

The Wnt/β-catenin pathway acts as a modulator of repair in cases where the pulp is exposed in the tooth (Figure 1). This pathway activates the local stem cells, thus expressing an early response to injury in various tissues, including the dental pulp [11,12,13]. When activated, it instrumentally participates in Dental Pulp Stem Cell (DPSC) proliferation and differentiation, augmenting the tissue repair and regeneration process [2]. The significance of Wnt signaling can be perceived by its role in the regulation of pulp cells and odontoblast function, regulation of homeostasis, tooth embryogenesis in various stages, tissue maintenance, and wound repair [14].

This pathway is intricated and comprises 19 ligands, the Wnt modulator in surface ectoderm (WISE), Wnts, 10 Frizzled receptors, the co-receptors Frz and Lrp5/6, coactivators such as Norrin and R-Spondins, antagonists such as Wnt Inhibitory Factors (WIFs), Notum, the Dkks, sFRPs and sclerostin [15,16,17]. The Wnt signaling consists of two pathways, the canonical and non-canonical. The soluble Wnt ligands bind with Frz receptors, thus initiating its effects, resulting in β-catenin accumulation and its transfer into the nucleus, promoting downstream transcription in the canonical pathway [18]. Wnt-PCP and Wnt-Ca^2+^ pathways are included in non-canonical signaling, and they engage in bioprocesses, yet the fundamental mechanisms are still unknown [19]. 

Wnt proteins are lipid-modified, glycosylated, cysteine-rich secreted proteins weighing about 40,000 that control migration, differentiation, and cell division during the embryonic phase [20,21]. Recent studies on this pathway have revealed its function in the process of pulp repair [22,23]. The extracellular binding of Wnt ligands to odontogenic progenitor receptors leads to the intracellular transduction of signals. This, in turn, affects the division, differentiation, and migration of cells through either dependent or independent pathways of β-catenin [24,25,26]. In Human Dental Pulp Cells (HDPCs), *Wnt5a* plays a role in increasing cell movement [26,27]. *Wnt3a* and *Wnt6* promote the division of odontoblasts precursors, whereas Wnt6 also aids in the migration of HDPCs [22,28]. 

In various cell types, *Wnt7b* expresses the Wnt/β-catenin and β-catenin independent pathways and is involved in the proliferation of cells [29,30]. Mechanically, *Wnt10a* participates with cells to synchronize murine fibroblasts to differentiate. This decline causes inhibition of proliferation and impaired dentin formation of murine mesenchymal cells [31,32]. In dentin formation, *Wnt10a* acts as a signaling molecule in different studies, but its biological effects on DPSCs have not been rigorously demonstrated [33].

**Figure 2:**
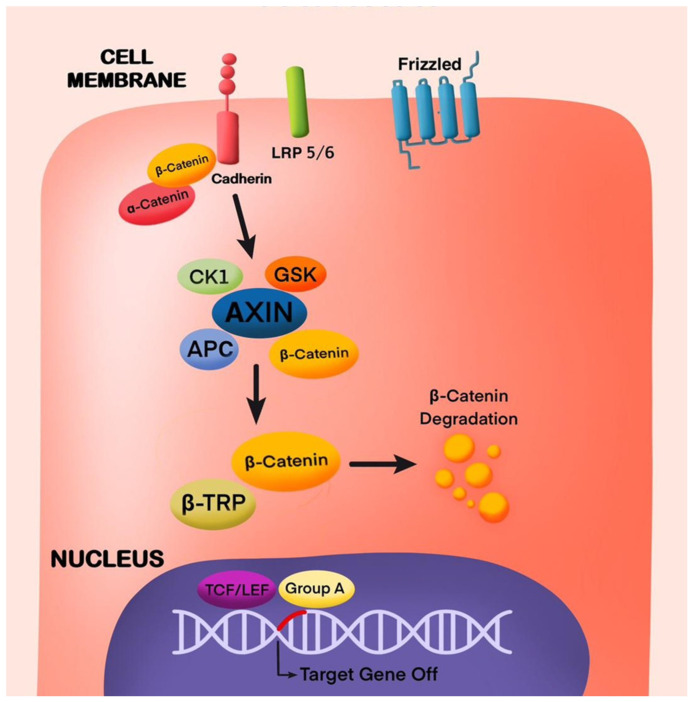
Inactive Canonical Wnt/β-Catenin Signaling Pathway. Cadherin activates *AXIN* mediated with the help of α and β-catenin and triggers gene expression.

**Figure 3:**
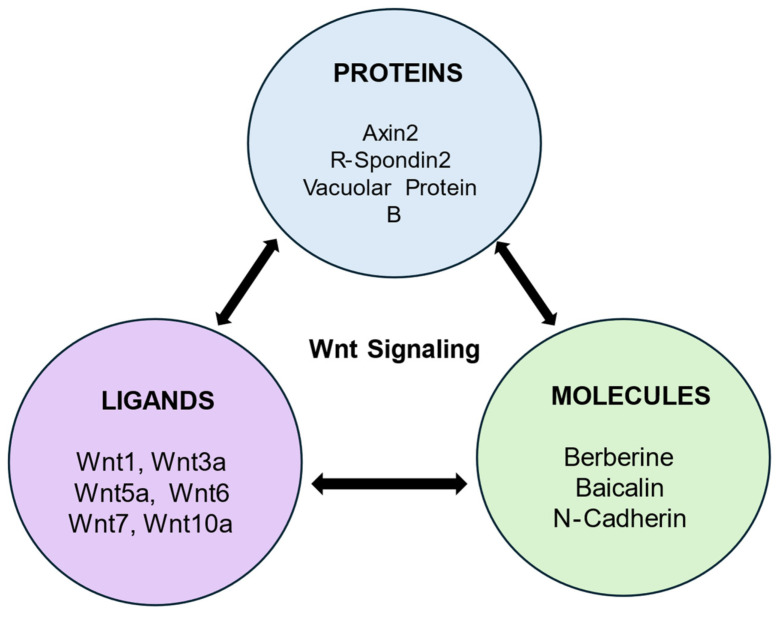
Different types of proteins, molecules, and ligands in Wnt signaling. The molecules activate Wnt signaling by modulating ligands (Wnt) and signaling proteins.

In the pulp repair process, TGFβ1 induces several biological effects by Wnt/β-catenin pathway stimulation [34]. Wnt-responsive genes such as *Axin2* play a role in maturation and tooth differentiation (Figure 2 and Figure 3). Inhibitors of GSK3β promote tertiary dentine production in vivo [23]. Cells expressing *Axin2* are an autocrine Wnt source, which differentiate and give rise to odontoblast-like cells that modulate tertiary dentinogenesis in response to injury [13]. In one study the regeneration as well as repair of teeth was stimulated by Lithium Chloride (LiCl) which activated the Wnt/β-catenin pathway. In injured pulp tissue, LiCl is applied to stimulate reparative dentinogenesis [35]. In another study, small molecule drugs such as tidiglusib, having anti-kinase activity were used to induce odontoblast differentiation [36,37]. Tideglusib, when incorporated in nanofibrous scaffolds is released in a controlled manner, stimulating DPSCs to proliferate and differentiate resulting in odontogenic differentiation and dentin regeneration [36,37]. This review highlights the recent advances in the Wnt/β-catenin signaling pathway in the oral cavity, how various factors and substances influence it, and its role in dentin repair and pulp regeneration.

## 2. Materials and Methods

### 2.1. Source of Data

**Figure 4:**
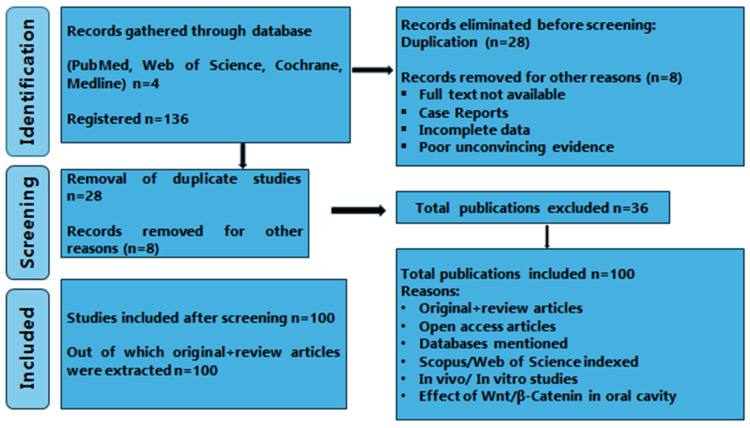
Prisma flowchart of recent advances in Wnt/β-Catenin pathway in dentin and pulp regeneration. Identification of articles through 4 platforms, followed by screening (duplicate and other reasons such as non-English articles) and then finalized 100 articles after screening for assessment.

The literature in this review is based on the PRISMA-DTA guidelines, as intimated in Figure 4. Different sources such as Scopus (https://www.scopus.com/home.uri), Medline (https://www.nlm.nih.gov/medline/medline_home.html), Cochrane (https://www.cochranelibrary.com/), and Pubmed Central (https://www.ncbi.nlm.nih.gov/pmc/?db=PMC) were used for this literature review. The selection of articles is based on a combination of mesh terms and keywords, as shown in Figure 4, based on publications from the last two decades (i.e., 2002 to 2023).

### 2.2. Search Method

Different keywords such as Wnt signals, dental pulp cells, β-Catenin, etc., and databases were searched for relevant publications. The included articles covered topics such as the Wnt signaling pathway, Dental Pulp Stem Cells (DPSC), the significance of Wnt and stem cells in pulp regeneration, β-catenin in the oral environment, and recent advancements in dentin and pulp regeneration. The total number of articles included was 136; out of that, 28 were removed because of duplication, so the total number was reduced to 108. Furthermore, 8 publications were omitted because of the unavailability of the full text, case reports, and partial data, as indicated in Figure 4. After careful selection, the articles were reduced to 100 following deduction. This systematic review relies on original studies conducted in vivo and in vitro, as well as multiple review articles, as depicted in Figure 4. The search strategies for the specific databases were customized and executed by merging MeSH terms, keywords, and phrases to obtain potential studies containing predictive mathematical equations. For instance, a search strategy for PubMed could be (“Dental Pulp Diseases”[Mesh] OR “Dental Pulp Calcification”[Mesh]) AND (“Dental Pulp/abnormalities”[Mesh] OR “Dental Pulp/chemistry”[Mesh] OR “Dental Pulp/cytology”[Mesh] OR “Dental Pulp/drug effects”[Mesh] OR “Dental Pulp/growth and development”[Mesh] OR “Dental Pulp/immunology”[Mesh] OR “Dental Pulp/injuries”[Mesh] OR “Dental Pulp/pathology”[Mesh]) AND (“Dental Pulp Diseases/drug therapy”[Mesh] OR “Dental Pulp Diseases/epidemiology” OR “Dental Pulp Diseases/immunology”) AND (“Wnt Proteins”[Mesh] OR “beta Catenin”[Mesh]) OR (“Wnt Proteins/adverse effects”[Mesh] OR “Wnt Proteins/pharmacokinetics”[Mesh] OR “Wnt Proteins/therapeutic use”[Mesh]) AND (“β-Catenin/pharmacology”[Mesh]). The following MeSH terms, keywords, and phrases were used in Scopus and Web of Science search: (“Dental tissue”) AND (“Wnt signal” OR “β-catenin signal”) AND (“Dental Pulp Regeneration”) AND (“Regulatory signals” OR “Dentistry”) AND (“Bioactive materials”) AND (“Wnt Signals” OR “β-catenin signals”).

Inclusion Criteria

1. Related studies in Wnt/β-catenin signals
2. Human (in vitro) and animal (in vivo) studies.
3. Wnt/β-catenin in oral cavity-based publications.
4. Bioactive materials on dental pulp cells via Wnt signals

Exclude and elimination criteria

1. Unconvincing or poor evidence.
2. Case report studies
3. Incomplete text publications
4. Incomplete data

The meticulously examined articles unveiled the Wnt/β-Catenin signaling pathway in dentin and pulp regeneration. Specifically, they shed light on the impact of various proteins, molecules, and ligands on this pathway, whether it be enhancing or inhibiting its activity.

### 2.3. PICO Framework

Population, P: Population of interest is in vivo and in vitro studies targeting the Wnt/β-catenin signaling pathway in dentin and pulp repair.

Interest/Exposure, I: Interest is in observing the role of the Wnt/β-catenin signaling pathway in regenerative dentistry.

Comparator, C: There is no comparator.

Outcome, O: It is the association between the Wnt/β-catenin signaling pathway and various molecules, ligands, and proteins, its activation and upregulation promoting dentin repair and pulp regeneration.

The review was designed to answer this PICO question: How does the Wnt/β-Catenin pathway play a vital part in tooth development, maintenance, repair, and regeneration? P: Articles with in vitro and in vivo studies of dental regeneration were investigated; I: Intervention, Effect of Wnt/β-catenin in the oral cavity along with bio-(molecules)materials; C: The different results of bio-(molecules)materials in the Wnt/β-catenin signaling pathways to support the dental tissues were compared; O: Observation, in-vivo and in-vitro signaling experiments for the dental regeneration by controlling physiological activities such as growth, differentiation, and migration were observed.

## 3. Results

This systematic review includes mainly original articles, certain review articles, and in vivo and in vitro research publications that consist of a total of 100 articles, as shown in Figure 4. The evidence-based table in this systematic review is based on different articles in Table 1. Various researches emphasize the mechanism of the Wnt/β-catenin pathway and its significance in the growth and repair of dentin and pulp tissues, along with the impact of various factors on this pathway in the formation of reparative dentin and regeneration of pulp, as illustrated in Table 1. The articles published in the last two decades were selected in this review, as shown in Figure 4.

Canonical Wnt signals are specific spatiotemporally; their expression measured via *Wnt10a* and *DKK1* is not constant but changes as the pulp regenerates. *Wnt10a* and *DKK1* are inversely related, suggesting the intricate control mechanism of Wnt signals in the regenerated pulp. It may act as a biomarker for regenerated pulp [63]. The repair and regeneration of tissues is modulated by the Wnt/β-catenin pathway. Reparative dentin is secreted by odontoblast-like cells, which are differentiated from the *Axin2*-expressing cells. In tertiary dentinogenesis, *Axin2* stimulates and modulates the Wnt/β-catenin pathway as a response to the injured tooth, providing a source of Wnt for regeneration [13]. Stathmin, in combination with *Wnt5a* in hDPSCs by the Wnt pathway, regulates the division and odontoblastic/osteogenic differentiation, thus playing a role in tooth and pulp regeneration [64].

In addition to its function in tooth development, this pathway promotes the growth of reparative dentin. In this study, GSK3β antagonists support the normal mechanisms of reparative dentin production in rat models to entirely regenerate dentin. Wnt signaling-related medicines are now perfectly suited for clinical therapy in dentistry because of this innovative technique [19]. *Wnt3a* promotes tertiary dentin formation by enhancing *OSX*, *BMP2*, and *DMP1* expression while reducing SHEDS colony formation resulting in increased mineralization. In *Wnt3a*-treated defects, a noticeable increase in dentin/bone volume is seen as dentin bridge formation; thus, it can be utilized in vital pulp therapy [60]. Small molecule inhibitors of GSKβ, such as c-MET inhibitors, activate the Wnt pathway, resulting in the repair of dentin and pulp regeneration in the exposed tooth [37]. 

In this research, the activation of Wnt signaling was achieved through the use of *Wnt3a* in the damaged tooth pulp. This led to the differentiation of pulp cells into secretory odontoblasts, which produced more tertiary dentin and ultimately improved the vitality of the pulp. This approach shows promise in the treatment of dental caries [28]. The migration of HDPCs and their ability to differentiate into odontoblasts were found to be enhanced by Wnt7b. This was achieved by stimulating the Wnt/β-catenin pathway and upregulating the JNK pathway. Therefore, Wnt7b could potentially be used as a therapeutic agent to improve the treatment of injured tooth pulp [42]. Furthermore, the expression of *Wnt10a* by odontoblasts was found to increase as the pulp regenerated, leading to dentin differentiation. This indicates that *Wnt10a* plays a crucial role in the regeneration of both dentin and pulp [62]. The positive regulation of DPSC proliferation and odontoblast differentiation through the *Wnt10a* pathway contributes to the repair process and is mediated by the Wnt/β-catenin pathway [2]. LiCl, by inhibition of GSKβ, activates the Wnt/β-catenin signaling pathway. LiCl augments the expression of *Axin2* and Wnt responsive cells, resulting in the differentiation of odontoblasts and increases the reparative formation of dentin when applied to dental pulp [55]. In *TCF/Lef: H2BGFP* reporter mice under the injury site, the odontoblasts and pulp cells responded to Wnt signaling, and the addition of tideglusib caused an increase in the Wnt signaling at the damaged pulp horn site. The findings demonstrated that injured dentin can be penetrated by small-molecule medications such as tideglusib, which then have an impact on odontoblasts and pulp cells, resulting in dentin and pulp regeneration [65]. 

Berberine in DPSCs promotes odontogenic differentiation by augmenting the nuclear β-catenin in the Wnt/β-catenin pathway; thus, it can be used to guide the treatment of dental caries [45]. Baicalin, a bioactive flavone acts as an anti-inflammatory, antioxidant, anti-tumor, and anti-viral agent. It inhibits NF-Kb and Wnt/β-catenin pathways, thus causing an increase in odonto/osteogenic differentiation of iDPSCs, resulting in pulp regeneration [61]. () Neuropilin 1, a multifunctional protein by stimulation of the Wnt pathway in DPSCs, plays a vital role in odontoblast differentiation and its mineralization, resulting in increased wound healing, tooth formation, and repair [66]. In hDPSCs, the *R-spondin2*, by activation and regulation of the Wnt/β-catenin pathway, encourages the proliferation together with odontogenic differentiation. The odontogenic activity of hDPSCs is impaired by *Rspo2* knockout. There is a synergistic effect of Wnt3a with *Rspo2* on Wnt/β-catenin signaling, thus providing an approach for reparative dentin formation [46]. 

Treated dentin matrix (TDM) in hDPSCs influences odontogenic differentiation by canonical Wnt/β-catenin pathway stimulation via GSKβ, resulting in dentin regeneration. TDM may be used as a base for regenerative tooth engineering [56]. Dentin secretion is modulated by the Wnt signaling pathway. Augmentation of Wnt signaling in pre-odontoblasts results in increased reparative dentin/osteodentin formation, thus contributing to vital pulp therapy by the formation of biologically based pulp capping material [44]. When MTA, calcium hydroxide, and Biodentine TM are applied directly to pulp, reparative dentin formation occurs at the exposure sites, thus promoting dentin bridge formation. Biodentine TM, when used in direct pulp capping, activates the Wnt/β-catenin pathway, resulting in increased reparative dentin formation [48]. 

Angelica sinensis polysaccharide (ASP) in human dental pulp stem cells (hDPSCs) through the Wnt/β-catenin pathway enhances cell proliferation and osteogenic differentiation, leading to increased dentin formation and pulp regeneration. Potential treatments for regenerative pulp diseases may involve ASP [67]. Treatment of DPSCs with lithium and *Wnt1,* followed by exposure to TEGDMA, triggers Wnt signaling, ultimately boosting pulp regeneration post-injury [41]. *Wnt3a*, *Wnt10a*, and β-catenin play a role in promoting reparative dentin formation from dental pulp cells and odontoblasts, facilitating the formation of dentin bridge [52]. Elevated blood glucose levels can induce cellular senescence and reduce pulp cell proliferation by upregulating β-catenin and *Wnt1* expression [57]. Activation of β-catenin by *Runx2* encourages dental pulp cells (DPCs) to differentiate into odontoblasts and deposit reparative dentin [40]. Exposure of pulp cells to *Wnt3a* leads to an increase in pre-odontoblasts and odontoblasts through the stimulation of the Wnt/β-catenin pathway, which is potentially useful in vital pulp therapy and dentin regeneration [68].

## 4. Discussion

### 4.1. Wnt/β-Catenin Pathway in Odontogenesis

**Figure 5:**
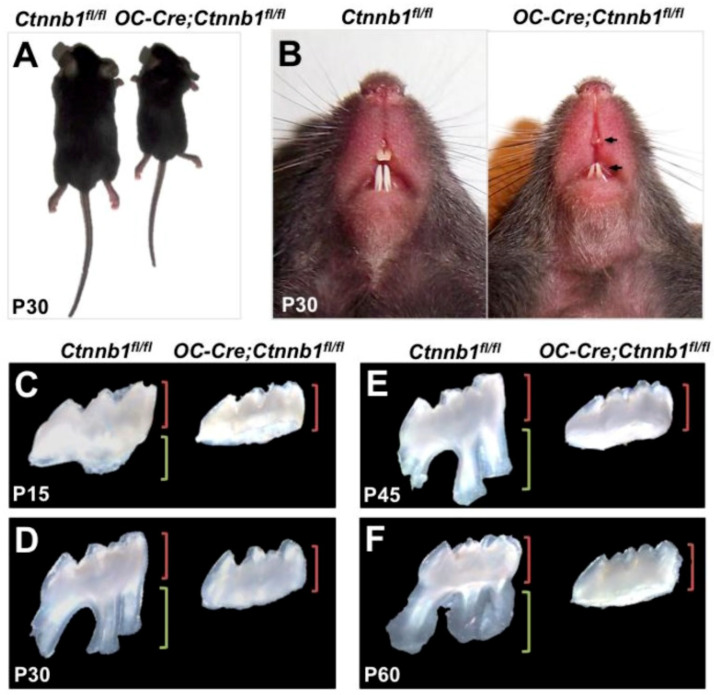
Growth delay and anomalies in the root of *OC-Cre, Ctnnb1 fl/f* mice (**A**) 1 month-old *OC-Cre, Ctnnb1 fl/f* mice are smaller than (*Ctnnb1 fl/fl*) the wild type mice. (**B**) Incisors at P30 show hypoplastic teeth with semi-transparent dentin (Gross appearance) (**C**–**F**). Maxillary 1 molars at P15, P30, P45, and P60 with lengths of crown and root are shown by green and red brackets, respectively [39]. Reproduced with permission from Zhang R et al. [39], published by International Journal of Biological Sciences, 2013.

In tooth and root odontogenesis, the role of the Wnt/β-catenin pathway is essential; thus, several studies have been conducted [19,40,46,53,56,59]. In one study, the β-catenin in odontoblast and cementoblast was removed [40]; this severely disrupted the molar root shape and formation and root analog of incisors. First, in vivo evidence was given in odontoblasts; this pathway is important in tooth root development, the differentiation and proliferation of cells, and the formation of root dentin. In *OC-Cre; Ctnnb1fl/f* mice, β-catenin withdrawal from pre-odontoblasts resulted in the silencing of differentiation of odontoblasts and reduced proliferation cellularly, resulting in the loss of tooth root due to arrested root dentin synthesis (Figure 5). In crown odontoblasts, which reach maturation in the embryo, the silencing of β-catenin had no visual impact on the maintenance and maturation. These data indicate the significance of Wnt/β-catenin signaling in root development by modulating the mesenchymal differentiation into odontoblasts, as shown in Table 1 [39]. 

As shown in Table 1, the Wnt/β-catenin signaling pathway has an important role in root formation, as demonstrated by the conditional β-catenin inactivation in immature odontoblasts [28,40,44,62]. This alteration results in the development of molars without roots [35]. It is imperative to exert precise control over Wnt signaling during root formation. In this study, areas were identified exhibiting Wnt/β-catenin signaling activity during the initial postnatal stages in the underlying mesenchyme and in the enamel knot. This may be due to the Wnt ligands and β-catenin expression postnatally in the coronal epithelium [69]. Notably, postnatally, when epithelial β-catenin was removed, ectopic incisors characterized by their short and malformed enamel were seen in the experimental mice. During the transition stage of root formation, heightened Wnt/β-catenin signaling in periodontal ligament cells, odontoblast-lineage cells, and HERS cells was perceived [70]. The Wnt/β-catenin signals were also detected in PN7 from the apical papilla and dental sac [54]. 

Odontoblast function is regulated essentially by the Wnt/β-catenin pathway [28,71]. In pulp, as age progresses, there is a decrease in Wnt signal and Wnt responsive cells because of a decrease in dentin secretion. Primary odontoblasts and their secretion of copious amounts of dentin have a huge role in maintaining the vitality of the pulp. However, the repair response reduces with age and results in chronic inflammation, leading to pulpal necrosis. Many endodontic cases are due to chronic trauma to the pulp [44]. A strategy was proposed to enhance the Wnt signal by delivery of a Wnt protein liposomal formulation to stimulate the repair potential of adult pulp [72] and dentin deposition (Table 1) [44]. 

### 4.2. Wnt Ligands and Their Role in Dentin and Pulp Regeneration

#### 4.2.1. *Wnt1*

**Figure 6:**
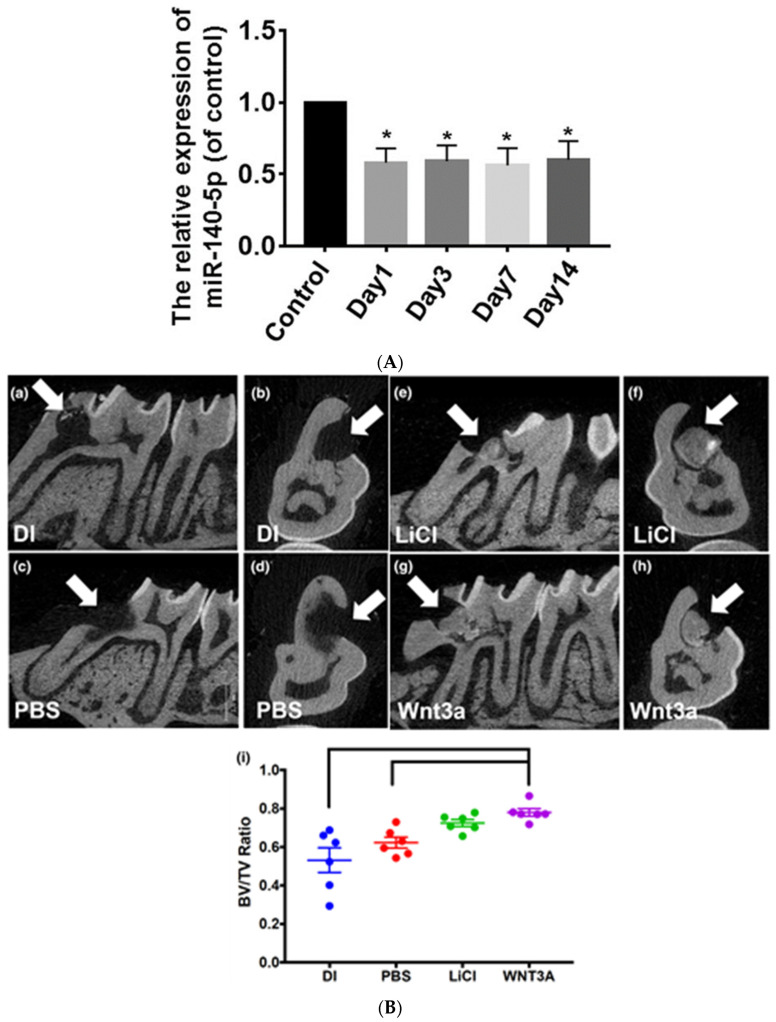
(**A**). Graph showing the decreased expression of miR-140-5p by qRT-PCR in differentiation groups on day 1, day 3, day 7, and day 14 vs the control group, *p* < 0.05. n = 6 whereby * represents significance of result [47]. Reproduced with permission from Lu Xiaohui et al. [47]., published by Stem Cell Research and Therapy (2019). (**B**). Tertiary dentin formation is promoted by Wnt3a in vivo in *Wistar* rats; molar injury as shown by white arrows in molars treated with distilled water (DI: (**a**,**b**)), phosphate-buffered saline (PBS: (**c**,**d**)), Lithium chloride (LiCl: (**e**,**f**)), and recombinant human Wnt3a (Wnt3a: (**g**,**h**)), (**i**) After 4 weeks analysis of specimens was performed. The dentin/bone volume to total volume was calculated as shown in the graph, with bars showing a significant difference [60]. Reproduced with permission from Sukarawan W et al. [60]., Published by International Endodontic Journal 2023.

As shown in Table 1, the Wnt/β-catenin pathway modulates DPSC differentiation via miR-140-5p. Moreover, the *Wnt1* gene in miR-140-5p was validated in this study. An increase in β-catenin levels in the inhibitor group and mimic group, a decrease was observed, thus showing the involvement of miR-140-5p in activating β-catenin. This study confirmed that miR-140-5p modulated the DPSC odontoblastic differentiation via the *Wnt1*/β-catenin signaling pathway. In DPSCs, the miR-140-5p expression decreases during the differentiation of odontoblasts as shown in (Figure 6A). This study indicates odontoblastic differentiation is supported by miR-140-5p inhibitors in DPSCs in vitro [47]. 

#### 4.2.2. *Wnt3a*

According to this study, when exposed pulp was treated with *Wnt3a*, there was an increase in the formation of tertiary dentin in comparison with the deionized water (DI) and phosphate-buffered saline (PBS) treated control groups in a rat model. This result confirms that *Wnt3a* can be used as a bioactive capping material for pulp to enhance reparative dentine formation. Lithium chloride-treated teeth also demonstrated dentine formation and a higher dentine/bone proportion than the total tissue proportion in comparison with the control group; however, no statistical difference was observed as shown in (Figure 6B) [55]. *BMP2* was identified as an inducing factor responsible for mRNA expression of odonto/osteogenic differentiation of SHEDS via the canonical Wnt signaling pathway. A significant increase in *BMP2* and mRNA levels was observed when treated with *Wnt3a*, and in osteogenic medium, calcium deposition of SHEDs was also observed. Thus, *Wnt3a* can be used as a pulp capping agent as it enables reparative dentine formation [60]. 

#### 4.2.3. *Wnt3a* and *Wnt10a* in Dentin Bridge Formation

As shown in Table 1, *Wnt3a* and *Wnt10a* are the ligands of Wnt, which, together with β-catenin, stimulate the canonical Wnt signaling [33,60,62]. Near the pulp capping area, on the 4th day of direct pulp capping, *Wnt3a* and *Wnt10a* were perceived. On the 4th, 7th, and 14th days, β-catenin was expressed strongly in the odontoblast cell nucleus. On the 28th day in the nucleus, a low positive signal was observed. The results of this study state that by autocrine process, *Wnt3a* and *Wnt10a* initiate the differentiation of reparative odontoblasts to produce dentin matrix. This supports the findings that odontoblasts and DPCs during dentin bridge formation secrete *Wnt3a* and *Wnt10a* [52].

#### 4.2.4. *Wnt10a*

This study shows the role of *Wnt10a* in dentin repair, where its overexpression in DPCs promotes cell proliferation and prevents odontoblastic differentiation. Immunostaining and reverse transcription PCR analysis showed the presence of *Wnt10a* in the cytoplasm of the odontoblast. Its activity, along with other Wnt molecules, was seen in cultured DPCs [33]. *Wnt10a* and *LEF1* were expressed before *DSPP* at the early stage of dentin formation [31,73]. The expression of *Wnt10a* enhances the differentiation and self-repair ability of stem cells at the early stage of dentin repair, as shown in Table 1 [33]. 

#### 4.2.5. *Wnt10a*, a Non-Cellular Agent for Dentin Pulp Regeneration

As shown in Table 1, an agent that can induce dentin formation by regulation of odontoblasts for dental pulp regeneration was developed. In this study, the hDPSCs were induced to differentiate into dentin. The expression of *DSPP*, *Wnt10a,* and *DKK1* on days 3 and 7 of induction were analyzed. On day 7, there was an increase in *Wnt10a* and *DSPP*, whereas *DKK1*, an antagonist of *Wnt10a*, decreased. On day 3 of transplantation, blood vessels, along with other fibrous tissues, regenerated in the root canal on day 3 after transplantation. There was no change in pulp content at days 3 and 7 post-transplantation, but odontoblasts increased, suggesting the maturation of dental pulp several days after transplantation. A consequential increment of *Wnt10a*-positive cells was seen along with *DSPP*. The increase in *DSPP*-positive cells from day 3 to 7 states the involvement of *Wnt10a* in the differentiation of odontoblasts rather than directly in pulp regeneration. In conclusion, the repair is augmented in pulp with an increase in *Wnt10a*, which acts as a non-cellular inducing agent (Table 1) [62]. 

#### 4.2.6. *Wnt7b*

This study shows the role of *Wnt7b* in the treatment of pulp injury by stimulating the HDPCs to proliferate and migrate via the Wnt/β-catenin and JNK signaling pathways [42]. In this study, an inhibitor of the JNK pathway *SP600125* is used to counteract the upregulation of HDPCs induced by *Wnt7b*, which shows the role of the JNK pathway. According to unpublished data, the levels of mineralization in *17IIA11* odontoblast-like cells (*A11*) increased by *Wnt7b* than in HDPCs. A11 differentiates into odontoblast-like cells, which secrete dentin matrix and are easily found in the late stage of odontogenic differentiation. This shows that *Wnt7b* is more efficient at this stage, which results in an increase in DSPP, which is a late odontogenic marker. As *Wnt7b* stimulates the proliferation and migration of HDPCs, it has an important role in the treatment of tooth injuries, such as dental trauma and dental caries, as well as in the reservation of vital pulp [42]. 

#### 4.2.7. *Axin2*

**Figure 7:**
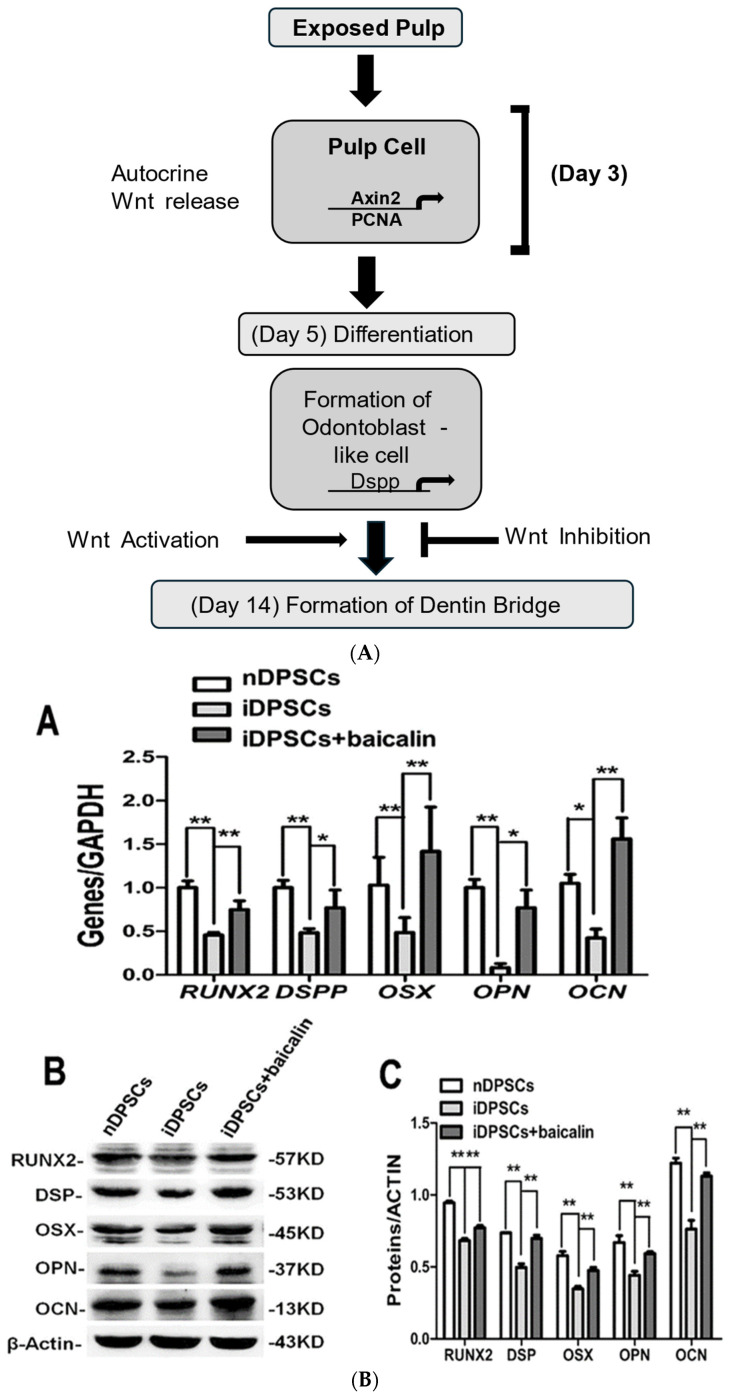
(**A**) The Wnt/β-catenin pathway regulates reparative dentinogenesis, which is modulated by the autocrine release of Wnt by *Axin2.* The rapid proliferation of pulp cells is perceived after tooth damage, with a peak at day 3 and a return to baseline on day 14, with dentin bridge formation. (**B**) The Wnt/β-Catenin signaling pathway is inhibited by Baicailin. (**A**,**B**) β-Catenin Protein expression of Baicailin treated iDPSC at each time point (**C**), The expression of *NLK*, *CaMK2*, β-catenin, and GSK3β of baicailin treated iDPSCs by western blot whereby the * and ** represent the significance of the result [61]. Reproduced with permission from Mengyuan Li et al. [61], published by Springer Nature 2023.

As illustrated in Table 1 [13], the utilization of *Axin2LacZ* and *TCF/Lef: H2B-GFP* reporter mice, coupled with real-time qPCR analysis for the *Axin2* expression in response to injury, disclosed a swift upregulation of Wnt/β-catenin signaling. The results from genetic tracing unveiled that cells expressing *Axin2* undergo a proliferative expansion after damage, with some of these cells differentiating into odontoblast-like cells. *Axin2* is expressed by post-mitotic primary odontoblasts, and an increase in Wnt/β-catenin signaling in *Axin2LacZ/LacZ* mice enhances reparative dentine production. The specific inhibition of *Axin2*-expressing cell’s ability to release Wnt had a profound impact on the formation of reparative dentin, emphasizing the crucial role of *Axin2*-expressing cells as the internal source of Wnt. This suggests that reparative dentin formation is orchestrated by autocrine Wnt/β-catenin signaling within *Axin2*-expressing cells themselves as shown in (Figure 7A). The identification of *Axin2* expression in odontoblast-like cells further supports the notion that these cells serve as the origin of their proliferative signals in the context of reparative dentinogenesis, as depicted in Table 1 [13]. 

#### 4.2.8. *R-Spondin 2*

As shown in Table 1, this study demonstrates the molecular mechanism of odontoblastic differentiation mediated by *Rspo2* by the activation of the Wnt signaling pathway. *Rspo2*, in combination with *Wnt3a*, augmented this pathway by expression of β-catenin. This study concludes that *Rspo2* operates synergistically with *Wnt3a* in the activation of this pathway for odontogenic differentiation and the formation of reparative dentin. The levels of *Wnt* and *Rspo2*-related receptors of *shRspo2* and *shNC* cells were compared in this study. *Rspo2* binds to the LGR4 receptor for the regulation of the Wnt/β-catenin pathway. The decreased expression of LRP5 and LRP6 in *shRspo2* activated through different Wnt ligands results in the decline in the Wnt/β catenin pathway activation [46]. 

### 4.3. Bioactive Molecules and Their Role in Dentin and Pulp Regeneration

#### 4.3.1. Baicalin

In this study, inflammatory dental pulp stem cells were isolated from inflamed pulp. Both inflammatory (iDPSCs) and normal (nDPSCs) stained positively for vimentin. In iDPSCs, TNF-α and IL-1β increased, while in baicalin-treated iDPSCs they decreased (Figure 7B). A concentration of 20 µM of baicalin facilitated iDPSCs to differentiate osteo/odontogenically with no change in proliferation. In DPSCs, the proliferation increased, and the differentiation decreased with inflammation. Therefore, a specific concentration of baicalin has an anti-inflammatory action in the pulp. In iDPSCs, β-catenin increases while *CaMKII* and *NLK* decrease. In the baicalin-treated group, there was a decrease in proteins augmented by *DKK1*. In baicalin-treated iDPSCs, the odonto/osteogenic differentiation was enhanced by inhibition of the Wnt/β-catenin pathway; thus, it may prove to be effective in repair in early pulpitis as shown in Table 1 [61]. 

#### 4.3.2. Berberine

Berberine is an important molecule in medicinal chemistry and pharmacology; it is a non-basic, quaternary benzylisoquinoline alkaloid [74]. The results showed that berberine increased the odontoblast differentiation marker proteins through the classical Wnt/β-Catenin signaling pathway and accelerated DPSC to differentiate odontoblastically. DPSCs were cultured from caries-free teeth and subjected to odontoblast differentiation. On day 14, the odontoblast differentiation of DPSCs was stained with alizarin red S and ALP, revealing that DPSCs were treated with berberine with *DKK-1* during the process of odontoblast differentiation. The nuclear β-catenin suppressed the *DKK-1* induced by berberine, while berberine caused the nuclear β-catenin to increase. Additionally, *Runx2* and *DMP-1*, which are downstream proteins of Wnt/β-catenin signaling, were activated at higher levels by berberine. In summary, Table 1 illustrates that berberine may be a novel medication for the treatment of dental defects [45]. 

#### 4.3.3. S-PRG Fillers as Dental Pulp Capping Agents

Lithium ions were added to S-PRG fillers to increase their effectiveness as dental capping agents, as Table 1 illustrates. S-PRG fillers were paired with a low concentration of LiCl in the pulp capping studies, and complete dentin production resulted. When compared with the other groups, it was discovered that from S-PRG/Li-100 mM samples, eluates could considerably augment cell migration, differentiation, and mineralization. In this investigation, at 7 and 14 days after the teeth were sealed with S-PRG cement without lithium in undamaged teeth, *Axin2* was significantly expressed in the S-PRG/Li-100 mM pulp-capped groups. The result of this study demonstrates that β-catenin expression was considerably elevated in the initial phases and precipitously decreased as *Axin2* continued to express itself [43]. 

#### 4.3.4. Vacuolar Protein 4B and Its Effect on the Wnt/β-Catenin Pathway

This study demonstrated the total cellular level of β-catenin was reduced by the decrease in vacuolar protein 4B (VPS4B). The nuclear translocation of β-catenin was negatively affected, while there was no change in the cytoplasmic concentration. LiCl, a GSK3β inhibitor by silencing VPS4B, partly resulted in odontogenic differentiation and proliferation, thereby causing the cellular β-catenin levels to rise. The Wnt/β-catenin signaling pathway promotes osteogenic proliferation and hDPSC differentiation. VPS4B silencing results in the retardation of hDPSC migration. Further research should be carried out to investigate the relationship between VPS4B and β-catenin signaling that further regulates the proliferation and differentiation of hDPSCs, as shown in Table 1 [49]. 

#### 4.3.5. N-Cadherin

As indicated in Table 1, an intentional reduction in N-cadherin in DPSCs resulted in a significant upregulation in β-catenin signaling, leading to increased odontogenic differentiation in vitro. This effect was nullified when cells were pre-treated with *XAV939*, a β-catenin inhibitor. Moreover, the silencing of N-cadherin facilitated the development of odontoblast-like cells, and a collagenous matrix was formed in β-TCP/DPSC composites transplanted into mice. Notably, the investigation revealed that the silencing of N-cadherin promoted the accumulation and translocation of β-catenin in the nucleus. Conversely, the inhibition of β-catenin by *XAV939* hindered the odontogenic differentiation process in DPSCs. The proposed mechanism suggests that N-cadherin silencing induces the translocation and accumulation of β-catenin in the nucleus. Subsequently, this promotes the binding of β-catenin to the *Runx2* promoter, facilitating *Runx2* and *DSPP* expression. Consequently, this sequence of events stimulates DPSCs to differentiate odontogenically. A downregulation of *Runx2* is required to achieve complete odontoblast differentiation in the context of dentinogenesis [53]. 

### 4.4. Lithium Chloride and Wnt/β-Catenin Pathway

As depicted in Table 1, a primary pulp culture derived from various reporter mice was utilized; this investigation delved into the mechanisms governing the effect of LiCl on the regulation of the differentiation of odontoblasts and osteoblasts. This study further demonstrated that the alterations induced by LiCl in the expression of *DSPP*, *DMP1*, and *BSP* in these early progenitors were effectively reversed by *DKK1* [55]. These findings strongly suggest the positive influences of LiCl on the differentiation of osteo/odontoblasts from αSMA+ progenitors via the Wnt/β-catenin signaling pathway [68,75]. This study indicated that prolonged and late exposure of dental pulp cells to LiCl resulted in an elevated expression of odontoblast markers facilitated by Wnt/β-catenin signaling. This exposure further increased the number of odontoblasts expressing *DMP1-Cherry* and *DSPP-Cerulean* transgenes. It is noteworthy that the inhibitory effects observed with prolonged exposure to LiCl differed from the stimulatory effects elicited by Wnt3a on pulp cells [55]. 

In another study, cells from tooth apical papillae (SCAP) with specific markers *CD24 STRO-1* and *CD146* were taken. The viability of SCAP increases when exposed to lower concentrations of LiCl (5 and 10 mM). Higher concentrations of LiCl (20 and 30 mM) inhibited cell viability by causing G2/M cell cycle arrest. After induction of mineralization differentiation (days 4–14), an increase is seen in *DSPP*, *ALP*, and *OCN*, i.e., the mineralized matrix had dentine matrix characteristics. A decrease in Wnt signaling and block of odonto/osteogenic differentiation is seen with *DKK1* addition, thereby preventing any Wnt1-mediated odontogenic events. Data support the significant promotion of SCAP differentiation and proliferation by Wnt/β-catenin, as shown in Table 1 [38]. 

### 4.5. Treated Dentin Matrix and Its Effect on hDPSCs via Wnt/β Catenin Pathway

The TDM (treated dentin matrix) extract is recognized for its richness in growth factors, including *TGFβ*, *DMP-1*, and *DSPP*, which possess the capability to induce the differentiation of stem cells odontogenically. In the TDM-induced odontogenic induction in human dental pulp stem cells (hDPSCs), various concentrations of TDM (10%, 20%, 40%, 60%, 80%, and 100%) were tested. The outcomes revealed a substantial increase in the odontogenic protein levels and mRNA in GSK3β-RNAi combined hDPSCs following TDM extract induction, as compared with the control group. Conversely, overexpressed GSK3β combined with hDPSCs, after TDM extract induction, resulted in reduced expression levels of *RUNX2*, *ALP*, *DSPP*, and *DMP-1*. These findings underscore the pivotal role of the Wnt/β-catenin signaling pathway in GSK3β-mediated odontogenic differentiation of hDPSCs [56]. The activation of the canonical Wnt/β-catenin pathway hinges on the stabilization and protection of β-catenin, achieved by inhibiting GSK3β [76]. This process facilitates the accumulation of β-catenin in the nucleus, where it collaborates with lymphoid enhancement factors and T cell factor (LEF/TCF) to exert its effects [77]. Additionally, a decrease in GSK3β expression during the odontogenesis of hDPSCs induced by TDM was observed, further emphasizing the involvement of the Wnt/β-catenin signaling pathway in this GSK3β-mediated odontogenic process as shown in Table 1 [56]. 

### 4.6. Role of Wnt Signalling in Progression of Diabetic-Induced Cellular Aging

As shown in Table 1, the effect of high glucose was investigated in the pulp in vitro model. To induce a diabetic state, solutions of concentrations 20 mM and 30 mM were prepared. The control was taken as 5 mM in a cell culture medium. After 2–3 weeks, the results showed that the glucose-enriched culture medium had an inhibitory effect on the proliferation rate. Compared with the control, there is an increase in beta galactosidase-positive cells in 20 mM and 30 mM groups. CDK inhibitor p21 expression increases, which is a cellular senescence marker. This study confirmed that chronic exposure to hyperglycemic environments induced senescence and cell cycle arrest [57]. In this study, *PNU74564* was applied, which inhibited Wnt signaling [78]. The results showed that β-catenin inhibition resulted in a decrease in senescent cells; however, LiCl increased senescence due to glucose. Therefore, Wnt signaling is a potential goal for the inhibition of senescence in hyperglycemic conditions, which suggests that bioactive materials should be developed specifically for pulp capping in diabetic patients [57]. 

### 4.7. Role of Wnt Pathway in Dentin and Pulp Repair

As shown in Table 1, repair in adult pulp may be stimulated by Wnt signaling. This is dependent on odontoblasts maintaining Wnt signaling in adulthood. Two approaches were used to prove adulthood Wnt responsive status in odontoblasts and pulp cells. First, analysis of frozen tissue section from adult *Axin2LacZ/+ Wnt* reporter mice was performed in which *X-galþve*. pulp cells and polarized odontoblasts were found. Second, *Axin2CreERT2/+*; *R26RmTmG/+* Wnt reporter mice showed *GFPþve* polarized odontoblasts. In L-PBS-treated samples, porous osteodentin is formed. In *L-Wnt3a* cases, *DSPþve* secretory odontoblasts formed reparative dentin, thus protecting the pulp. Based on the pulp cell’s reliance on Wnt signaling, a new technique for root canal therapy was evaluated. The liposomal *Wnt3a* protein improved the healing of pulp, thus giving an approach to achieving pulp regeneration in humans [28]. 

Materials such as calcium hydroxide and MTA are used to treat pulp exposure because of the treatment of caries. The evaluation revealed that tertiary dentin was similar to natural dentin. To induce reparative dentinogenesis, a controlled therapeutic amount of material was applied locally to prevent the negative effect of these small molecular Wnt agonists. GSK3β, an inhibitor, gives rise to reparative dentine at defect sizes translatable to minor human lesions, but it is not clear how to fully “regenerate” the lost tissue [50]. Reparative dentine is produced to heal the wound after contact with exposed pulp and pulp capping material. Pulp capping materials such as MTA and Biodentine TM have shown high clinical success rates compared with calcium hydroxide. Biodentine TM treatment showed better results when compared with MTA. On histological analysis, there was complete bridge formation with no inflammation after Biodentine TM capping. The study results also suggest the stimulation of the Wnt/β-catenin pathway by Biodentine TM. In pulp inflammation, *SDF1* causes hDPSC migration. These data suggested all capping materials, calcium hydroxide, MTA, and Biodentine TM stimulate cell cycle progression [79,80,81]. Wnt signaling plays a role in Biodentine TM-induced reparative dentine formation as β-catenin was expressed in the Biodentine TM-treated group, as shown in Table 1 [48]. 

In another study, the endogenous canonical Wnt signaling pathway was activated by alternate mechanisms in DPSCs. For indirect Wnt activation, either lithium (LiCl) is used [82], and for direct, human recombinant (hr) *Wnt-1* is used [83]. LiCl concentration causes a stimulatory and inhibitory effect on cells, indicating that increased GSK3β inhibition may be toxic to cells [84]. DPSCs, when exposed to monomer released by TEGDMA, activated the Wnt/β catenin signaling. During the repair of pulp, stem cells have a vital role in tissue engineering as well as the development of new bioactive materials that can cause repair via Wnt signaling [41]. 

Wnt signaling plays a crucial role in controlling the epithelial–mesenchymal transition (EMT) process through various mechanisms. Notably, the inhibition of GSK3β via multiple pathways, including the Wnt cascade, leads to the stabilization of β-catenin and Snail. This stabilization, in turn, triggers cell migration, proliferation, and the development of cancerous growth. Consequently, GSK3β emerges as a pivotal regulator of epithelial organization and function, presenting itself as a potential molecular target for therapeutic interventions.

## 5. Conclusions

The Wnt/β-catenin signaling pathway, encompassing both canonical and non-canonical components, is crucial for the growth, maintenance, restoration, and rejuvenation of teeth. It facilitates the development of teeth, cementum, dentin production, and the formation of periodontal tissues. Abnormalities in tooth development arise from either excessive or insufficient activity of the Wnt signaling pathway. The activation of the Wnt/β-catenin signaling pathway leads to the maturation of odontoblasts that produce tertiary dentin, aiding in the repair of damaged dental pulp and enhancing tooth vitality. Calcium hydroxide, MTA, and Biodentine TM promote pulp healing by inducing reparative dentin formation. Biodentin TM showed β-catenin expression, thus suggesting Wnt signaling pathways part in the repair of dentin and pulp. Pulp repair and regeneration are further improved by the introduction of various Wnt ligands and molecules, which may enhance the effect of the Wnt/β-catenin signaling pathway. Increased understanding of the Wnt/β-catenin signaling pathway in dentin and pulp repair and regeneration has led to the discovery that it plays a role in promoting pulp vitality, treating dental caries, and providing an alternative approach for root canal treatment. The major limitation is the inadequate comprehension of numerous natural bioactive substances when it comes to effectively regulating Wnt signals in dental applications. Further research on the Wnt/β-catenin signaling pathway may provide a path for therapeutic and regenerative dentistry.

## Figures and Tables

**Table 1:** Evidence-based table of Recent Advances in Wnt/β-Catenin Signaling Pathway in Dentin and Pulp Regeneration.

S	Year	Author	Type of Study	Sample Size	Objective	Conclusion	Recommendation/Limitation
1	2012	J Wang et al. [38]	In vitro	n = 8 3 molars 18–20 age	Role of Wnt/β-catenin signaling pathway in SCAP proliferation and differentiation.	Proliferation and odonto/osteogenic differentiation of SCAP are promoted by the Wnt/β-catenin signaling pathway.	Further study in SCAP mineralization and proliferation may bring improvement in dental tissue engineering.
2	2013	Ran Zhang et al. [39]	In vivo		Wnt signaling and its importance in cementogenesis and odontogenesis.	Wnt signaling encourages the differentiation of odontoblasts from mesenchyme.	Influence of Wnt signaling on tooth development.
3	2014	Zichun Zhang et al. [33]	In vivo	9	Response of *Wnt10a* on DPSCs.	Proliferation of DPCS is promoted, and their odontoblastic differentiation is negatively regulated by *Wnt10a*.	Further research is vital to understand the biological effect of *Wnt10a* on DPCS.
4	2014	Nana Han et al. [40]	In vivo		β-catenin during the activation of *Runx2* in DPCs augments odontoblastic differentiation.	Odontoblastic differentiation in tertiary dentin modulated by β-catenin.	The mechanism of reparative dentin formation remains unclear.
5	2015	Danial J Hunter et al. [28]	In vivo	72 mice	Response of injured pulp to amplified Wnt signaling resulting in superior pulp healing.	Amplified Wnt signaling caused pulp cells to differentiate into secretory odontoblasts, thus improving pulp vitality.	The role of amplified Wnt signaling on pulp cells plays a role in promoting pulp vitality, treating dental caries, and alternative approaches for root canal treatment.
6	2015	Athina Bakopoulou et al. [41]	In vitro	3 molars age 18–24. DPSCs cultured from 3 donors.	The function of the Wnt/β-catenin pathway in the healing of pulpal injury resulted from resinous monomers via DPSC mediation.	DPSCs, under the effect of Wnt/β-catenin, participate in pulp healing after injury.	The analysis of stem cells is important for pulp regenerative therapy and bioactive molecule planning via tissue engineering through Wnt activation.
7	2016	Masato Tamura et al. [15]	Review Article		Determination of function of signaling molecules of Wnt in tooth.	Tooth development, maintenance, and turnover are regulated by both canonical and non-canonical Wnt pathway.	The Wnt signaling pathway and its progress in future therapeutics for tooth regeneration.
8	2017	Hongyang LV et al. [42]	In vivo/In vitro	HDPCs cultured from 3 molars from 19 to 24 years of age.	Function of Wnt7b protein in HDPCs relocation and differentiation.	Wnt/β-catenin and JNK pathways partly have a role in the differentiation of HDPCs and are promoted by the Wnt7b protein.	Wnt7b can be used in the potential treatment of dental caries and tooth trauma. Wnt7b plays a role in maintaining the vitality of the tooth.
9	2017	Rebecca Babb et al. [13]	In vivo		The role of *Axin 2* via Wnt/β-catenin signaling in tertiary dentinogenesis.	As a response to tooth injury, new odontoblast-like cells are formed via the Wnt signaling pathway, and *Axin 2* cells provide a signal in reparative dentinogenesis.	Further study is required for stem cell-based tooth repair via the Wnt/β-catenin signaling pathway in secondary and tertiary dentinogenesis
10	2018	Manahil Ali et al. [43]	In vivo/ In vitro	21	To check the enhancement of S-PRG cement through the addition of LiCl and its effect on hDPSCs.	S-PRG cement through the Wnt/β-catenin pathway enhances reparative dentin formation in rat teeth and promotes hDPSC profiles.	Inclination of reparative dentinogenesis in clinical trials needs to be further assessed. The interaction of Li ions with S-PRG fillers is not fully understood.
11	2019	Yuan Zhao et al. [44]	In vivo	2 strains of *Wnt reporter* mice	The function of Wnt signaling in age-linked variations in pulp and its ability to respond to subacute traumas.	Amplification of Wnt signaling results in reparative/osteodentin organization, as the secretion of dentin from odontoblasts is regulated by this pathway.	Wnt signaling and its role in dentin secretion can be used to form biological pulp-capping material.
12	2019	Xi Lu et al. [19]	Review Article		Review of Wnt signaling pathway, its modulators, and their function in odontogenesis and therapeutic potential.	Tooth development is closely regulated by the canonical Wnt pathway.	The tooth regeneration method may be provided by a detailed study in Wnt signaling.
13	2019	Anqian Wu et al. [45]	In vitro	(N) n = 20 pulp Ages 18–25	Impact of berberine on DPSCs and their odontoblast differentiation.	By the stimulation of the Wnt pathway, berberine encourages odontoblast differentiation.	Berberine might be used as a new drug for the treatment of dental defects.
14	2019	Yuping Gong et al. [46]	In vivo	20	Role of *Rspo2* in promoting hDPSCs to differentiate odontogenically through the Wnt/β-catenin pathway.	hDPSCs stimulated by *Rspo2*, both positive and negative, by enhancer and silencer agents, thus enhancing their proliferation and differentiation.	Future work of *R-spondins* and Wnt ligands could be on therapeutic use clinically.
15	2019	Xiaohui Lu et al. [47]	In vitro	n = 10 3 molars age 14–22 yrs	miR-140-5p impact on the odontoblastic differentiation of DPSC.	The odontoblastic differentiation of DPSCs is regulated by lowering miR-140-5p by targeting the Wnt/β-catenin pathway.	miR-140-5p can be used as a medicinal agent that could control odontoblastic differentiation in dental medicine.
16	2019	K. Yaemkleebua et al. [48]	In vivo	32 rat molar pulps were used.	Hard tissue formation after application of pulp capping materials on pinpoint exposure and the role of Wnt signaling and cell cycle regulation.	Reparative dentinogenesis occurred with the application of pulp capping materials, and cyclin D1 expression was seen.	The association between Wnt signaling and reparative dentin formation might be used to comprehend the response of normal pulp in injured conditions.
17	2019	Yuhua Pan et al. [49]	In vivo In vitro	DPSCs culture from 3 molars of 18–20 yr Donors	The role of VPS4B in DPSCs via the Wnt/β-catenin signaling pathway.	DPSC differentiation and proliferation are regulated by VPS4B via the Wnt/β-catenin pathway.	Dentin dysplasia type1 could be treated via potential medicinal therapies by the formation of dentin and reparative endodontic therapy.
18	2020	LK Zaugg et al. [50]	In vivo/ In vitro	Adult male *Wistar* rats (7 wk old) and CD1 mice (6 wk old)	The Wnt/β-catenin signaling and its function in reparative dentinogenesis and how it is affected by the introduction of GSK3β inhibitor drugs.	GSK3β inhibitor results in the tertiary dentin formation at the defect region having the size of a human lesion; however, this is not true regeneration.	Augmentation of reparative dentinogenesis can be used potentially as a treatment in direct pulp capping.
19	2021	Nicha Tokavanich et al. [51]	Review Article		Wnt signaling and its effect on postnatal tooth development.	Wnt signaling may play a vital role as a target for root formation and tooth eruption disorders.	In-depth study of the Wnt signaling pathway may provide a path in the future for regenerative dentistry.
20	2021	Miroku Hara et al. [52]	In vivo	321 mice	Dentin bridge formation at pinpoint exposure and how the Wnt signaling and its molecules affect it.	The injured dental pulp is repaired by the stimulation of canonical Wnt signaling.	Wnt pathway activation by the dental pulp capping materials may be a new approach for vital pulp therapy.
21	2021	Zilong Deng et al. [53]	In vitro/In vivo	3 molars Pulp 18–25 yr n = 5 mice	N-cadherin effect on DPSCs, in-vitro and in-vivo, and its function in odontogenic differentiation.	N-cadherin negatively regulates β-catenin activity in DPSCs.	Further study is required on how neurogenic differentiation of DPSCs is regulated by N-cadherin
22	2021	Jia Wang et al. [54]	In vivo	2 transgenic mice	Role of Wnt signaling and distribution of *BMP* during postnatal root development.	Tooth root development is regulated by *BMP* and Wnt signaling pathway.	Further study is required for Wnt/β-catenin pathway regulation for matrix secretion and differentiation in postnatal tooth development.
23	2021	Anushree Vijaykumar et al. [55]	In vitro	Pulp culture from 1 and 2 molar 5–7 d mice	Dentin pulp complex and role of LiCl in its reparation and regeneration via Wnt/β-catenin pathway by inhibiting GSK3β.	LiCl results in reparative dentin formation, which has an improved structure.	GSK3β controls several transcription factors and various pathways; thus, the influence of LiCl on tertiary dentin formation may not be deemed exclusive to the Wnt/β-catenin pathway.
24	2021	Sirui Liu et al. [56]	In vitro In vivo	DPSCs culture donor’s teeth pulp 18–25 yr Wistar mice 26	Wnt/β-catenin signaling role in hDPSCs and its odontogenic differentiation via TDM extract.	TDM via GSK3β activates the Wnt/β-catenin pathway in hDPSC and stimulates its odontogenic differentiation.	The mechanism for TDM-induced differentiation requires further study.
25	2021	Mona Asghari et al. [57]	In vitro	4000 cells	Effect of hyperglycemia and function of Wnt pathway in senescence of pulp cells.	Hyperglycemia causes the aging of pulp cells. Cellular aging is induced via beta-catenin.	Further study is required to determine the role of beta-catenin and pulp regeneration in diabetic patients.
26	2022	Henry F Duncan et al. [58]	In vivo In vitro	MMP13 deficient mice and WT control mice were used for DPCs.	The effect and interaction of *MMP13* with the Wnt signaling pathway. Its effect on tooth development, repair, and dentin pulp regeneration.	*MMP13* controls the organization and regulation of tooth development in addition to dentin pulp regeneration.	This study found targets for new therapy for traumatized pulp by increasing repair response.
27	2022	Tingting Fu et al. [59]	In vitro	DPSCs Culture Donar teeth ≤16 yr	Role of SNHG1 in hDPSCs in odontogenic differentiation via Wnt/β-catenin signaling.	The Wnt/β-catenin pathway is silenced by IncRNA SNHG1 via miR-328-3p, resulting in the differentiation of hDPSCs.	Useful in regenerative endodontics.
28	2023	Waleerat Sukarawan et al. [60]	In vivo	6 mice	To check in SHEDs the influence of *Wnt3a* on the odonto/osteogenic differentiation and reparative dentin production.	*Wnt3a* causes an increase in osteogenic differentiation, silencing of proliferation in SHEDs, advances tertiary dentin formation, and can be used as a biological molecule in vital pulp therapy.	An in-depth study is required to identify the function and role of *Wnt3a* in tissue engineering.
29	2023	Mengyuan Li et al. [61]	In vitro	DPSC In 6 well plates	Effect of Baicalin on DPSC differentiation via Wnt/β/NF-kB pathway.	Baicalin impedes both NF-Kb and Wnt/β-catenin pathways, thus promoting the differentiation of DPSCs, causing repair of pulp with early irreversible pulpitis.	Further investigation and research are required to apply it in the long-term
30	2023	Shintaro Sakatoku et al. [62]	In vitro	DPSC in 24 well plates	Role of *Wnt10a* and odontoblasts in regenerative dental pulp.	Odontoblasts expressing *Wnt10a* play an increased role in pulp regeneration, with dentin-inducing capacity.	A detailed study after transplantation is required to check the long-term effect of *Wnt10a* along with *DKK1* levels in regenerated dental pulp.

## Abbreviations

Wnt—Wingless-related integration site; DPSC—Dental pulp stem cells; iDPSC—Inflammatory Dental pulp stem cells; DKK-1—Dickkopf-1; TGFβ1—transforming growth factor beta-1; SCAP—Stem Cells From the Apical Papilla; S-PRG—surface pre-reacted glass ionomer; VPS4B—Vacuolar Protein Sorting 4 Homolog B; MMP13—Matrix Metallopeptidase 13; SHED—stem cells from human exfoliated deciduous teeth; OSX-Osterix; cMET—Mesenchymal Epithelial Transition Factor; ASP-Angelica Sinensis Polysaccharide; Lef1—Lymphoid Enhancer Binding Factor; CaMK2—Calcium Calmodulin Dependent Protein Kinase-1; NLK-Nemo like Kinase; OCN-Osteocalcin; TNF-α—Tumor Necrosis Factor; BMP1—Bone Morphogenetic Protein-1; FRZ—Frizzled Receptors; nDPSC—Normal Dental Pulp Stem cells; HDPC-Human Dental Pulp Cells; GSK3β—Glycogen synthetase kinase 3 beta; JNK—c-Jun N-terminal Kinase; Rspo-2—R-Spondin-2; TDM—Treated Dentin Matrix; SNHG1—Small Nucleolar RNA Hose Gene 1; NF-fB pathway—Nuclear Factor pathway; DMP1—Dentin Matric Phosphoprotein 1; MTA—Mineral Trioxide Aggregate; TEGDMA—Triethylene Glycol Dimethacrylate; DSPP-Dentin Sialophosphoprotein; ALP-Alkaline Phosphatase; BSP-Bone Sialoprotein; CDK—Cyclin Dependent Kinase; IL-1 β—Interleukin 1 beta.
